# HIV testing service awareness and service uptake among female heads of household in rural Mozambique: results from a province-wide survey

**DOI:** 10.1186/s12889-015-1388-z

**Published:** 2015-02-12

**Authors:** Heather N Paulin, Meridith Blevins, John R Koethe, Nicole Hinton, Lara ME Vaz, Alfredo E Vergara, Abraham Mukolo, Elisée Ndatimana, Troy D Moon, Sten H Vermund, C William Wester

**Affiliations:** Department of Medicine, Division of Infectious Diseases, Vanderbilt University School of Medicine, 1611 21st Avenue South, A-2200, Medical Center North, Nashville, TN USA; Department of Biostatistics, Nashville, TN USA; Department of Pediatrics, Nashville, TN USA; Department of Preventive Medicine, Nashville, TN USA; Vanderbilt University, Vanderbilt Institute for Global Health (VIGH), Nashville, TN USA; Friends in Global Health (FGH), Maputo, Mozambique; Save the Children, Washington, D.C. USA

**Keywords:** HIV/AIDS, Voluntary Counseling and Testing (VCT), Resource limited settings, Southern Africa, Mozambique, Rural, HIV awareness

## Abstract

**Background:**

HIV voluntary counseling and testing (VCT) utilization remains low in many sub-Saharan African countries, particularly in remote rural settings. We sought to identify factors associated with service awareness and service uptake of VCT among female heads of household in rural Zambézia Province of north-central Mozambique which is characterized by high HIV prevalence (12.6%), poverty, and suboptimal health service access and utilization.

**Methods:**

Our population-based survey of female heads of household was administered to a representative two-stage cluster sample using a sampling frame created for use on all national surveys and based on census results. The data served as a baseline measure for the *Ogumaniha* project initiated in 2009. Survey domains included poverty, health, education, income, HIV stigma, health service access, and empowerment. Descriptive statistics and logistic regression were used to describe service awareness and service uptake of VCT.

**Results:**

Of 3708 women surveyed, 2546 (69%) were unaware of available VCT services. Among 1162 women who were aware of VCT, 673 (58%) reported no prior testing. In the VCT aware group, VCT awareness was associated with higher education (aOR = 2.88; 95% CI = 1.61, 5.16), higher income (aOR = 1.41, 95% CI = 1.06, 1.86), higher numeracy (aOR = 1.05, CI 1.03, 1.08), more children < age 5 in the home (aOR = 1.53; 95% CI = 1.07, 2.18), closer proximity to a health facility (aOR = 1.05; 95% CI = 1.03, 1.07), and mobile phone ownership (aOR = 1.37; 95% CI = 1.03, 1.84) (all p-values < 0.04). Having a higher HIV-associated stigma score was the factor most strongly associated with being less likely to test. (aOR = 0.41; 95% CI = 0.23, 0.71; p<0.001).

**Conclusions:**

Most women were unaware of available VCT services. Even women who were aware of services were unlikely to have been tested. Expanded VCT and social marketing of VCT are needed in rural Mozambique with special attention to issues of community-level stigma reduction.

## Background

Despite global efforts to scale up human immunodeficiency virus (HIV) prevention measures and voluntary counseling and testing (VCT) for HIV, testing coverage remains low in many sub-Saharan African countries, particularly in rural areas [1-4]. Similar to many Sub-Saharan African countries, Mozambique has increased the number of total adults undergoing HIV testing (ages 15–49) between 2003–2009, although baseline testing rates (e.g., 2003 and years prior) were very low.

Thus, by 2009 only a minority of men (17.2%) and women (33.6%) reported they had ever received HIV testing and were aware of their status [5]. Programmatic analyses of VCT and treatment initiatives supported by the United States government-funded President's Emergency Plan for AIDS Relief (PEPFAR) have focused primarily on urban and peri-urban populations. Fewer such evaluations have been conducted among persons receiving HIV care and treatment in rural, more remote settings [6-14].

Mozambique is one of the most HIV-affected countries in sub-Saharan Africa, with an estimated national HIV prevalence of 11.5% (ages 15–49) and 1.2 million persons (age 15+) living with HIV in 2009 [15,16]. In 2004, the Mozambican Ministry of Health (MISAU) launched a national antiretroviral treatment (ART) program, termed “*tratamento antiretroviral*” (TARV). By 2009, ART services were offered in all 10 provinces and 1 capital city, but care was still predominantly clustered in urban areas [17-20]. The importance of increased VCT participation has intensified with the growing body of evidence that early ART initiation may be a highly effective method to reduce new infections (i.e., treatment as prevention) [21-24]. As of 2010, it was estimated that approximately 32% of adults in Mozambique with advanced HIV disease are receiving ART, but coverage remains highest in urban areas [16,18,25].

Zambézia is a predominantly rural province situated in north-central Mozambique, with a population near 3.8 million persons, making it the nation’s 2nd most populous province. Zambézia, has the 3rd highest HIV prevalence (among provinces) at 12.6% (ages 15–64) in 2009 [15], faces numerous challenges including exceedingly high poverty rates, suboptimal health service access and utilization rates, and poor ART coverage, with an estimated 10% ART coverage rate [15,18,26,27]. Other neighboring countries had low ART coverage rates, but Zambézia was certainly among the worst. The economy of Zambézia is primarily agrarian and piscatory, with only one large population center in Quelimane, the provincial capital (2007 census population of 193,343 persons) [28]. The national language is Portuguese and the five predominant local languages are Cisena, Elomwe, Echuabo, Cinyanja, and Emakhuwa [29]. The province has the highest unmet needs in terms of HIV/AIDS care treatment with recent statistics documenting that 20% of Mozambique’s persons living with HIV/AIDS live in Zambézia, with many of them residing in rural areas [15]. The province has a limited health infrastructure with 214 health facilities, including one provincial hospital and 6 rural hospitals. As of December 2012, it was estimated that only 33 of these health facilities were providing ART. We have calculated that if every HIV positive Zambézian (≈230,000 persons) were to be identified and enrolled in care, each health facility would need to provide care for an average of 7,000 persons [15,27].

The disproportionately higher burden of HIV disease among women in Mozambique is similar to the rest of southern Africa [19,30]. Furthermore, a 2010 report suggested that despite programs to increase VCT and treatment coverage, there has been no significant national reduction in the prevalence of HIV among females aged 15–24, in contrast to neighboring countries [20]. In Mozambique, Portuguese is the national language and VCT sites were first known as the *Gabinete de Aconselhamento e Testagem Voluntária* (GATV or Centres for Voluntary Counselling and Testing); they are now known as *Unidades de Aconselhamento e Testagem em Saúde* (UATS or Health Counselling and Testing Units). The motivation for changing the name in 2006–2007 was that GATV sites only tested for HIV. In an attempt to diminish stigma associated with attending a site known as the “HIV testing site” the UATS began providing counseling and testing for a variety of health issues including malaria, tuberculosis, family planning, as well as HIV. Access to VCT (UATS), provider-initiated counseling and testing (PICT), and ART services need expansion in rural Mozambique [31].

In this study, we describe factors associated with the women’s VCT service awareness and utilization across the province through a representative survey.

## Methods

The 5-year *S*trengthening *C*ommunities through *I*ntegrated *P*rogramming (SCIP) project, known locally as *Ogumaniha,* was initiated as a multi-faceted health and economic development initiative financed by the United States Agency for International Development (USAID) in Zambézia Province, Mozambique. As the monitoring and evaluation partner in the *Ogumaniha* project, Vanderbilt University and its local non-governmental organization, Friends in Global Health, conducted a baseline household survey in 2010 of more than 3,700 female heads of household throughout the province, including many remote communities.

### Sample design and data collection

**Figure 1:**
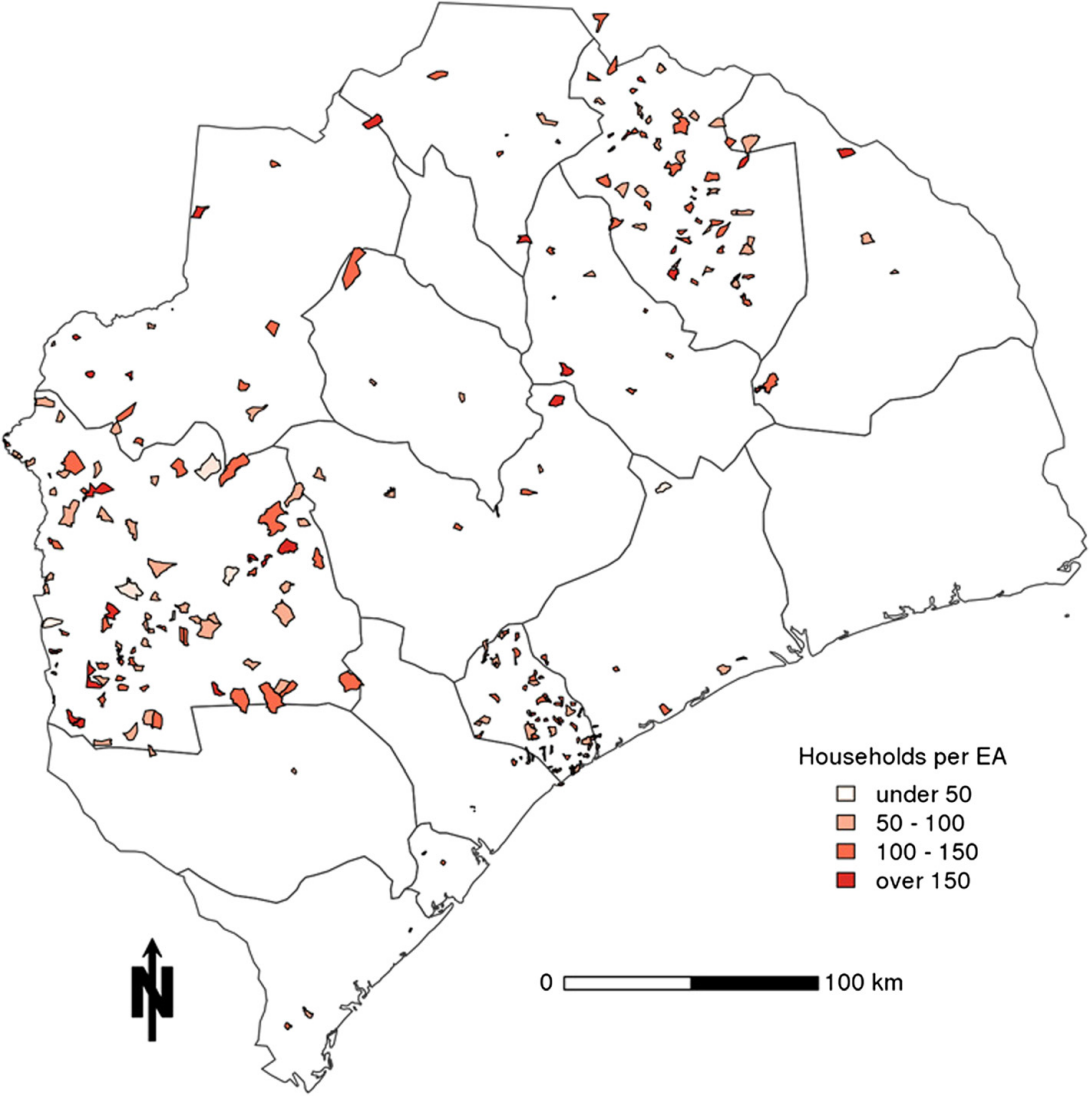
**Enumeration Areas surveyed for the** ***Ogumaniha*** **baseline survey, Zambézia, Mozambique 2010.**

Our two-cluster sampling design was executed by the Chief Sampling Statistician from the Mozambican National Statistics Institute (*Instituto Nacional de Estatística*). Also, our two-stage cluster sampling design referenced a 2007 census based sampling frame that was utilized for all national surveys. A provincially representative sample of 264 enumeration areas was selected, with probability proportional to size according to the census (Figure 1). Enumeration areas were stratified into urban and rural areas; 206 of the enumeration areas (EAs) were selected from three “focus districts” (Alto Molócuè, Morrumbala, and Namacurra), further stratified by planned intervention.

The baseline survey data were collected during August-September 2010. Local authorities were notified prior to the arrival of the survey team. From topographic maps, the survey teams divided the EAs into four quadrants. Starting in the center of the assigned quadrant, interviewers selected a direction, then chose the first household in this direction (i.e. starting point), and then approached the nearest four households for interview. This household selection approach was used following the failed attempt to use satellite imagery to list and randomly select households. Female interviewers conducted the survey with female heads-of-household, defined as the only or principal wife of the immediate family of the household.

The female head of household was chosen to be the respondent, as she is the most likely subject to be the most familiar with the health and care taking of the entire family. In polygamous families, the eldest wife was selected, which may have introduced bias if the remaining wives and their children were different.

Interviewers were trained to conduct interviews in Portuguese or in one of the five predominant local languages. Fourteen mobile teams, consisting of a team leader and four interviewers, administered the survey face-to-face using cell phones with an electronic questionnaire installed for data collection.

In cases where cell phone access was unavailable, the survey was administered face-to-face using a paper questionnaire and responses were later entered into the cell phone. Whenever possible, survey data were transmitted securely via a wireless network to a central server; when not possible, data were transferred by a backup system from cell phones to a location in Maputo, the nation’s capital, and then uploaded to the central server.

### Survey instrument

The *Ogumaniha* project was designed with a monitoring system to report on project performance indicators and included a baseline survey instrument. The survey was developed by a multi-disciplinary team of health, education, economics, and social science experts at University Eduardo Mondlane (UEM) in Maputo and Vanderbilt University in the USA. Based on the expert opinion of the team, questions and scales were chosen from previously validated national surveys in Mozambique and derived from human development theory [32]. Domains of inquiry for the 550 item survey instrument included demographics and child health information [33], literacy and numeracy [34], education achievement [35,36], agricultural practice, food security, dietary diversity and food coping strategies [37-39], material possession and consumption of goods presumed critical for well-being [40-44], income generation [45], social barriers and social participation, social support networks, decision making within the family and gender differences [46-49], self-agency and determination [50], reproductive health, malaria and HIV knowledge, attitudes and practices, HIV/AIDS stigma [51], and a numeracy score (range 0–57; with scores less than 16 indicating the ability to read individual letters only).

Three scales were employed in the analysis, all with a range of 0–100. Higher scores on the *Quality of Life (QoL) scale* reflect positive answers about short-term subjective well-being [52-54]. Higher scores on the *Social Exclusion Stigma scale* indicate a lack of willingness to support and/or interact with persons living with HIV/AIDS (PLWHA) [55]. Higher scores on the *Negative Labeling and Devaluation Stigma Scale* indicate a negative judgment and/or stereotype PLWHA, including anticipating devaluation by peers if one were to become infected with HIV oneself [55]. Hence, a high QoL scale score suggests high QoL while high scores of the two stigma scales are both suggesting more stigma.

### Ethical review board oversight

The study protocol was reviewed and approved by the National Committee of Bioethics for Health in Mozambique and the Institutional Review Board of Vanderbilt University. No incentive was provided to participants, and written informed consent was obtained for all study participants. A mark was permitted if a woman could not sign her name.

### Statistical considerations

Estimates of descriptive statistics were weighted by the inverse of the household sampling probability. Continuous variables are reported as weighted estimates of the median (interquartile range). Categorical variables are reported as weighted percentages. Two multivariable logistic regression models were used to determine the relationship between specific variables (selected *a priori*) and awareness of HIV VCT service availability (*model 1*: unaware vs. aware of VCT and *model 2*: aware but never tested vs. aware and ever tested in a VCT setting).

Only respondents who answered if they had ever heard of VCT were included in the analysis. From 550 variables, and we pre-specified the following 18 variables of interest: age, years of formal education, distance to the nearest health facility, urban or rural residence, primary language of household, understanding of Portuguese, marital status, religion, household autonomy, number of children less than 5 years of age, income, household electricity, mobile phone ownership, literacy, numeracy, willingness to stigmatize and exclude people living with HIV/AIDS (PLWHA) using each stigma scale, and QoL.

To relax linearity assumptions, some continuous variables were included in the models using restricted cubic splines. Multiple imputation was utilized to account for missing values of predictors and to prevent case-wise deletion of missing data. We used the functions ‘*aregImpute*’ and ‘*fit.mult.impute*’ from the Hmisc package in R-software 2.15.1 (www.r-project.org) that used predictive mean matching to take random draws from imputation models; ten imputation data sets were used in the analysis. We fit generalized linear models with robust covariance matrix estimates to correct for correlated responses among households from the same enumeration area [56].

## Results

We interviewed 3,749 female heads of household utilizing 14 survey teams over 49 days (≈77 interviews per day, median interview time 73 minutes) with a participation rate of 99.7%. Among these, 3,708 (98.9%) responded to questions on VCT knowledge and were included for analysis. Table 1 summarizes demographic information for the women who were head of household respondents. The median age of the group was 29 years (range: 16 to 90). Women had a median 2 years of formal education (range: 0 to 15). Most households (80%) were located in rural areas; the median distance of each enumeration area to the health facility was 6.6 kilometers. Only 39% of women reported understanding Portuguese with 8% reported it as their primary language. The predominant religion was Catholicism (45%). Most women (74%) were married or in a common-law relationship. When asked who was the household decision maker regarding pregnancy health care and child health care, 67% stated that this was women together with men.

**Table 1:** **Respondent and household characteristics by knowledge of volunteering counseling and testing from** ***Ogumaniha*** **baseline survey, Zambézia, Mozambique 2010**

	**Unaware of VCT**	**Aware of VCT**	**Combined**
	**(n = 2546)**	**(n = 1162)**	**(n = 3708)**
**Age of respondent in years (range)** ^**a**^	30 (23 – 38)	27(23–35)	29 (23–37)
**Education in years (range)** ^**a**^	0 (0 – 3)	3 (0 – 5)	2 (0–4)
**Distance of Enumeration Area from health facility in km (range)** ^**a**^	7.6 (4.4 –11.3)	5.0 (1.0 – 9.3)	6.6 (3.3 - 10.3)
**Urban/rural** ^**b**^			
Rural	90%	66%	80%
Urban	10%	34%	20%
**Primary language of household** ^**b**^			
Cinyanja	12%	20%	15%
Cisena	18%	6%	13%
Echuabo	24%	23%	24%
Elomwe	44%	34%	40%
Emakhuwa	<1%	<1%	1%
Portuguese	2%	17%	8%
**Respondent understands Portuguese** ^**b**^	33%	48%	39%
**Marital status** ^**b**^			
Married/Common Law	75%	73%	74%
Divorced/Separated	4%	4%	4%
Single	17%	17%	17%
Widowed	4%	6%	5%
**Religion** ^**b**^			
Catholic	44%	45%	45%
Protestant	14%	16%	15%
Evangelical and Pentecostal	17%	16%	16%
Other Christian^c^	3%	7%	4%
Muslim	10%	7%	9%
Non-Christian Eastern	3%	2%	2%
Other^c^	9%	7%	8%
**Decision maker for pregnancy health care** ^**b**^			
Both	52%	57%	54%
Men	18%	13%	16%
Women	30%	31%	30%
**Decision maker for child health care** ^**b**^			
Both	66%	70%	67%
Men	16%	11%	14%
Women	18%	20%	19%

Legend:

^a^Continuous variables are reported as weighted estimates of median (interquartile range), with each observation being weighted by the inverse of the household sampling probability.

^b^Categorical variables are reported as weighted percentages, with each observation being weighted by the inverse of the household sampling probability.

^c^‘Other Christian’ includes Latter day Saint/Mormon and Jehovah’s Witness. ‘Other’ includes Spiritual, Traditional Religions, and Agnostic or Atheist.

Fewer women were aware of VCT (31% unweighted; n = 1162) than were unaware (69% unweighted; n = 2546; Table 1). Women unaware of VCT had lower median formal education compared to those aware of VCT (0 vs. 3 years), lived further from the health facility (7.6 vs. 5.0 kilometers), and more frequently resided in rural areas (90% vs. 66%). In addition, women unaware of VCT less frequently understood Portuguese (33% vs. 48%) and less frequently reported Portuguese as the primary language spoken in their households (2% vs. 17%).

### Aware of VCT vs. unaware of VCT

In the first model we identified numerous factors that were associated with *being aware of VCT* services in the province (Table 2). Older age was associated with lower probability of VCT awareness. Compared with women who were 50 years of age, 30 year old women had the highest likelihood of being aware (Adjusted Odds Ratio [aOR] = 1.47; 95% confidence intervals [CI] = 1.12, 1.93). Compared with no formal education, women having received >10 years of formal education had a nearly 3-fold higher odds of being aware that VCT services were available (aOR = 2.88; 95% CI = 1.61, 5.16). Residing in an urban compared to a rural setting also increased one’s likelihood of being aware of VCT (aOR = 2.31; 95% CI = 1.45, 3.68). For every 1 kilometer women resided closer to a health facility, the odds of VCT awareness increased by 5% (aOR = 1.05; 95% CI = 1.03, 1.07). Understanding Portuguese was not a strong predictor of VCT awareness (p = 0.4). However, female heads of household residing in homes where Portuguese was the primary spoken language had 2.5 times higher odds of VCT awareness (aOR = 2.53; 95% CI 1.37, 4.70) compared to Elomwe (the most frequently spoken language among surveyed participants), and approximately twice the odds of VCT awareness compared to homes where Cinyanja and Echuabo were the predominant spoken dialects (adjusted odds ratios of 2.13 [95% CI = 1.39, 3.28] and 1.83 [95% CI = 1.38, 2.43], respectively).

**Table 2:** **Multivariable logistic regression models: aware of VCT and ever tested in VCT**

	**Adjusted odds ratio for aware of VCT**	**P-value**	**Adjusted odds ratio for ever tested in VCT**	**P-value**
	**(95% CI)**		**(95% CI)**	
**Number of households in model**	**3708**		**1162**	
**Age (years)**		0.02^a^		0.53
20	1.10 (0.82, 1.47)		1.15 (0.75, 1.77)	
30	1.47 (1.12, 1.93)		1.10 (0.82, 1.47)	
40	1.18 (1.01, 1.38)		1.05 (0.91, 1.21)	
50 (ref)	1		1	
65	0.85 (0.55, 1.31)		0.93 (0.75, 1.16)	
**Education (years)**		<0.01^a^		0.21
0 (ref)	1		1	
1	0.99 (0.89, 1.09)		1.04 (0.98, 1.12)	
2	1.00 (0.83, 1.20)		1.09 (0.95, 1.25)	
5	1.32 (0.98, 1.79)		1.24 (0.88, 1.75)	
10	2.88 (1.61, 5.16)		1.54 (0.78, 3.05)	
**Distance to Clinic (per 1 kilometer decrease)**	1.05 (1.03, 1.07)	<0.01	1.00 (0.97, 1.03)	0.96
**Urban**	2.31 (1.45, 3.68)	<0.01	1.36 (0.91, 2.04)	0.13
**Primary Language**		<0.01		0.32
Elomwe (ref)	1		1	
Cinyanja	2.13 (1.39, 3.28)		1.11 (0.56, 2.20)	
Cisena	0.83 (0.62, 1.13)		0.92 (0.59, 1.45)	
Echuabo	1.83 (1.38, 2.43)		0.75 (0.53, 1.06)	
Emakhuwa	1.02 (0.31, 3.35)		0.36 (0.04, 3.20)	
Portuguese	2.53 (1.37, 4.70)		0.60 (0.33, 1.09)	
**Understands Portuguese**	1.10 (0.89, 1.35)	0.38	1.29 (0.95, 1.75)	0.12
**Marital status**		0.63		0.21
Married/Common Law (ref)	1		1	
Single	0.89 (0.70, 1.12)		1.35 (.98, 1.86)	
Divorced/Separated	0.79 (0.52, 1.12)		0.80 (0.41, 1.55)	
Widowed	0.95 (0.65, 1.38)		1.21 (0.68, 2.17)	
**Religion**		0.99		0.64
Catholic	1		1	
Protestant	1.04 (0.77, 1.40)		1.26 (0.80, 1.98)	
Evangelical and Pentecostal	1.05 (0.79, 1.40)		1.23 (0.78, 1.96)	
Other Christian^c^	1.03 (0.66, 1.61)		0.86 (0.45, 1.65)	
Muslim	1.01 (0.73, 1.40)		0.86 (0.54, 1.38)	
Non-Christian Eastern	1.13 (0.62, 2.06)		1.41 (0.60, 3.32)	
Other^c^	1.16 (0.84, 1.60)		1.02 (0.59, 1.76)	
**Decisions made for seeking health care for a pregnancy**		0.88		0.77
Both (ref)	1		1	
Men	1.01 (0.74, 1.37)		1.24 (0.68, 2.27)	
Women	1.06 (0.84, 1.34)		1.08 (0.75, 1.56)	
**Decisions made for seeking health care for a child**		0.21		0.31
Both (ref)	1		1	
Men	0.75 (0.54, 1.05)		1.00 (0.56, 1.77)	
Women	1.04 (0.81, 1.33)		0.76 (0.53, 1.09)	
**Children Under 5 years of age**		0.02		0.02^b^
None (ref)	1		1	
2	1.24 (1.03, 1.48)		1.63 (1.12, 2.36)	
4	1.53 (1.07, 2.18)		1.29 (0.95, 1.75)	
**Monthly Income**		0.04		0.33
No Income (ref)	1		1	
1-1000 Meticais	1.19 (0.99, 1.43)		1.17 (0.80, 1.70)	
>1000 Meticais (> ≈ $1.00 USD/day)	1.41 (1.06, 1.86)		1.37 (0.90, 2.08)	
**Electricity**	0.95 (0.59, 1.55)	0.85	0.98 (0.57, 1.67)	0.94
**Mobile Phone**	1.37 (1.03, 1.84)	0.03	1.21 (0.77, 1.89)	0.4
**Literacy (per 1 point)**	1.00 (0.99, 1.00)	0.36	1.00 (0.99, 1.01)	0.61
**Numeracy (per 1 point)**	1.05 (1.03, 1.08)	<0.01	1.02 (0.98, 1.06)	0.34
**Negative Labeling and Devaluation Stigma Scale (0–100)**		0.73		0.02^b^
0	0.98 (0.88, 1.10)		0.91 (0.58, 1.42)	
20 pts (ref)	1		1	
40	1.02 (0.91, 1.15)		1.06 (0.80, 1.39)	
60	1.04 (0.82, 1.32)		0.76 (0.51, 1.13)	
80	1.07 (0.75, 1.52)		0.41 (0.23, 0.71)	
**Social Exclusion Stigma Scale (0–100)**		<0.01^a^		0.89
0	0.54 (0.45, 0.65)		0.99 (0.88, 1.11)	
20 pts (ref)	1		1	
40	1.21 (1.08, 1.34)		1.01 (0.90, 1.13)	
60	0.82 (0.65, 1.04)		1.02 (0.81, 1.28)	
80	0.61 (0.47, 0.79)		1.02 (0.73, 1.45)	
**Wellbeing (Chronic quality of life score)** (per 10 points)	1.06 (1.00, 1.13)	0.05	0.94 (0.87, 1.02)	0.16

Legend:

^a^There is evidence that age, education, and social exclusion stigma scale are non-linear with aware of VCT; these variables are modeled using restricted cubic splines.

^b^There is evidence that number of children under 5 and ‘Negative Labeling and Devaluation Stigma Scale’ are non-linear with ever tested in VCT; these variables are modeled using restricted cubic splines.

^c^‘Other Christian’ includes LDS Mormon and Jehovah’s Witness. ‘Other’ includes Spiritual, Traditional Religions, and Agnostic or Atheist.

Compared to women with no children, women with 2 children under 5 years of age were more likely to be aware of VCT (aOR = 1.24; 95% CI = 1.03, 1.48). Having even more children was associated with an even heightened awareness of VCT (e.g., four vs. no children, aOR = 1.53; 95% CI = 1.07, 2.18). Women residing in a household where the income was $1.00 USD/day or higher had 41% higher odds of VCT awareness (aOR = 1.41, 95% CI = 1.06, 1.86). We did not detect an association between women residing in households where electricity was available versus not, but mobile phone ownership was associated with increased awareness of VCT (aOR = 1.37; 95% CI = 1.03, 1.84). Additionally, a non-linear relationship of social exclusion stigma was noted with VCT awareness, such that persons having very low levels of social exclusion stigma or with moderate to high levels had the lowest odds of VCT awareness. Having a higher numeracy score was also significantly associated with having heightened awareness of VCT (aOR = 1.05, CI 1.03, 1.08); specifically, for every 1-point increase in numeracy score, one’s odds of VCT awareness were increased by 5%. As well-being scores increased on the QoL scale, women were increasingly more aware of VCT, (aOR = 1.06; 95% CI 1.00, 1.13); specifically, for each 1-point increase in QoL score, the odds of VCT awareness increased by 6%. Little association with VCT awareness was noted for the Negative Labeling and Devaluation Stigma Scale (p = 0.7).

### Aware but never tested in VCT vs. aware and ever tested in VCT

In the second model among women *who were aware of VCT* (n = 1,162), 58% (unweighted n = 673) remained untested in a VCT setting, while 42% (unweighted n = 489) had tested previously. Among these VCT-aware women, women with 2 children were most likely to have tested (aOR = 1.63; 95% CI = 1.12-2.36 vs. 0 children). Having children under the age of 5 was the only covariate significantly associated with both being aware of VCT as well as being aware and undergoing VCT. Among VCT-aware women, those with high stigma scores (≥ 80 points on the Negative Labeling and Devaluation Stigma Scale) were 59% less likely to be tested (aOR = 0.41; 95% CI = 0.23, 0.71 [20 pts ref]; p<0.001) compared to those with lower stigma scores.

## Discussion

In this systematic, province-wide assessment of female respondents across Zambézia province, less than one-third of women were aware that VCT was locally available. Expanding this service awareness is a critical first step in the early identification of new HIV infections. Awareness of VCT was higher among those who were younger, resided in an urban area, and had greater socio-economic advantages (highly educated, primarily Portuguese speaking, relatively wealthy, and/or a mobile phone user). Awareness was also higher among participants residing in closer proximity to a health facility, reporting a higher quality of life, and having less stigmatizing attitudes. Being multiparous and caring for greater than or equal to 2 living children was highly associated with VCT awareness and uptake, suggesting that testing awareness might be increasing during and after pregnancy, presumably due to prevention of mother to child HIV transmission (PMTCT) services.

A successful strategy for VCT requires that testing is available, the public is aware of such services, and that a person is able to overcome potential barriers and attend the facility offering VCT [57]. While several prior studies investigated barriers to various forms of VCT access and factors related to the acceptance or refusal of these services in predominantly rural areas, our study looks further upstream and describes factors related to the actual awareness of the availability of these services (a necessary prerequisite to uptake) [2,9,58-62]. Given Mozambique’s historically low national rate of VCT uptake (14% for women in 2009) [16,63], compounded by Zambézia’s very high rates of illiteracy (50% among Portuguese speakers and 94% among non-Portuguese speakers) and their low numeracy levels (mean score corresponding to US kindergarten-level skills) [29], these findings suggest increased efforts are needed to expand basic awareness and knowledge related to VCT if service uptake is to be increased. Similar to our findings, a Tanzanian study found that only 45% of women in a rural cohort were aware of VCT services, a finding that was negatively associated with VCT uptake [4]. While Zambézia Province could benefit from enhanced efforts to expand knowledge and availability of VCT, outreach must be tailored to a local language that women understand.

Most women in Zambézia reside in remote, rural communities with pronounced socio-economic disadvantages. When women become pregnant in Zambézia, they are offered opt-out HIV testing during antenatal care (i.e. provider initiated counseling and testing). As women have more children their awareness and utilization of VCT seemed to have increased, independent of a woman’s age. This is due perhaps to their pregnancy-related exposure to the health care system. Several investigators reported a positive association of increased exposure to the health care system and VCT uptake [63-65]. As of 2010, the majority of Zambézian women did routinely access health facilities antenatally; however, nearly 40% did not go to the facility during their last pregnancy missing opportunities to receive HIV testing in a PMTCT setting [18]. In addition to scaling up antenatal services, we recommend integrating VCT services concurrently with postnatal care and childcare visits.

A very strong predictor of a woman being unaware of VCT was her stigmatizing view of PLWHA, supporting their social exclusion. Negative labeling and devaluation were predictors for being less likely to test. A refusal to test that is associated with higher stigma has been seen in other studies [64,66-69]. Campaigns designed to address and reduce stigma, if successful, may facilitate VCT uptake.

Strengths of our study include the representative reach of our survey, the access to remote rural areas typically neglected in research, the use of local languages to gather data, the comfort women had responding from the privacy of their homes, and the size of our sample in a remote, understudied setting. Limitations to our study include those familiar to a cross-sectional survey, including respondent reporting bias and the potential for miscommunication and misclassification of results due to variety of local languages and dialects in the province (though we interviewed in any of six languages). Household ownership of a mobile phone was ascertained via the respondent’s spouse’s possession of a mobile (not the respondent herself). We could not cross-reference VCT uptake self-reports against medical records. In addition, the VCT program in Mozambique changed its name in 2006–2007 to be less specific for HIV and to include a broader context of health issues in order to reduce stigma associated with testing for HIV; the survey referred to the old name and not the new one, though our interviewers do not believe that this affected responses.

## Conclusions

In summary, our survey of 3,708 female heads of household in rural Zambézia Province, Mozambique, found that a majority of women were unaware of VCT availability and most women who were aware of these services had not used them. Increased efforts are required to expand knowledge and the availability of VCT among women in this rural context; and ideally campaigns should be tailored to a woman’s specific sociolinguistic context. Rural community based testing and counseling via mobile brigades [59,70-75] and door-to-door campaigns [74,76-88] can be effective and could potentially impact Zambézia.

Another option may be incorporating such interventions into existing perinatal and post-natal healthcare visits where women are likely to attend for their child’s care. Finally, Provider-initiated counseling and testing (PICT) strategies should be promulgated far more aggressively to enable HIV-infected women to be identified and subsequently started earlier on ART.
